# The junctional epithelium originates from the odontogenic epithelium of an erupted tooth

**DOI:** 10.1038/srep04867

**Published:** 2014-05-02

**Authors:** Sara Yajima-Himuro, Masamitsu Oshima, Gou Yamamoto, Miho Ogawa, Madoka Furuya, Junichi Tanaka, Kousuke Nishii, Kenji Mishima, Tetsuhiko Tachikawa, Takashi Tsuji, Matsuo Yamamoto

**Affiliations:** 1 Department of Periodontology, Showa University School of Dentistry, 2-1-1 Kitasenzoku, Ohta-ku, Tokyo 145-8515, Japan; 2 Research Institute for Science and Technology, Tokyo University of Science, Chiba 278-8510, Japan; 3 Organ Technologies Inc., Tokyo 101-0048, Japan; 4 Division of Pathology Department of Oral Diagnostic Sciences, School of Dentistry, Showa University School of Dentistry, 1-5-8, Hatanodai, Shinagawa-ku, Tokyo, 142-8555, Japan

## Abstract

The junctional epithelium (JE) is an epithelial component that is directly attached to the tooth surface and has a protective function against periodontal diseases. In this study, we determined the origin of the JE using a bioengineered tooth technique. We transplanted the bioengineered tooth germ into the alveolar bone with an epithelial component that expressed green fluorescence protein. The reduced enamel epithelium from the bioengineered tooth fused with the oral epithelium, and the JE was apparently formed around the bioengineered tooth 50 days after transplantation. Importantly, the JE exhibited green fluorescence for at least 140 days after transplantation, suggesting that the JE was not replaced by oral epithelium. Therefore, our results demonstrated that the origin of the JE was the odontogenic epithelium, and odontogenic epithelium-derived JE was maintained for a relatively long period.

Periodontitis is a chronic inflammatory disease that is caused by oral bacterial infection and results in the progressive destruction of the supporting structure of teeth^1^. Recently, periodontitis has been reported to contribute not only to local destruction but also to systemic diseases, including cardiovascular disease, diabetes, arteriosclerosis, preterm low birth weight, and aspiration pneumonia^2^,^3^,^4^.

Generally, the epithelium, called the junctional epithelium (JE), is directly attached to the tooth surface (enamel) and has a defensive role against continuous bacterial infection. After bacterial pathogenic components in dental plaque, such as lipopolysaccharide, cause gingival inflammation, the defense system is destroyed; furthermore, the JE is detached from the tooth surface and transformed to the pocket epithelium, and a small area remains attached to the root (attachment loss). The periodontal tissue breakdown begins here. Therefore, the JE is involved in the pathogenic mechanism of periodontitis.

Histologically, although the gingival epithelium is keratinizing squamous epithelium, the JE is a non-keratinized squamous epithelium. The JE has been recognized as the first line of peripheral host defense against dental flora^5^. For example, epithelial cells constituting the JE have only a few desmosomes, which aid mononuclear leukocytes infiltration as compared with oral epithelium, which have abundant desmosomes^6^. In addition, the JE is known to express defensive factors against inflammation. For example, we previously reported that secretory leukocyte protease inhibitor (SLPI) and S100A8 are characteristically expressed in the JE. SLPI protects the intestinal epithelium from proteases secreted as part of the inflammatory response and is associated with the maintenance of tissue integrity^7^. S100A8 and S100A9 form a heterodimeric complex and constitute calprotectin, an antimicrobial peptide^8^. Furthermore, we reported the constitutive expression of chemokines and cytokines, such as keratinocyte-derived chemokine, macrophage inflammatory protein-2, and interleukin-1β, in the JE^9^. Moreover, the developmental and morphological features of the JE and oral epithelium have been shown to be different, suggesting that they have different origins. Several studies have reported that the JE originates not from the oral epithelium but from the reduced enamel epithelium, which is the odontogenic epithelium that remains around the enamel surface of an erupting tooth^10^,^11^,^12^,^13^,^14^,^15^,^16^. Similarly, *Nanci* et al. showed that both odontogenic ameloblast-associated and amelotin were expressed in the JE^17^,^18^,^19^. Therefore, it seems acceptable that the origin of JE is the reduced enamel epithelium at the initial stage of tooth eruption. However, whether the reduced enamel epithelium-derived JE is maintained for a lifetime without replacement by the oral epithelium remains controversial.

In the present study, we clarified the origin of the JE using a bioengineered tooth germ method^20^,^21^(Fig. 1a). Our results demonstrated that the origin of the JE was the reduced enamel epithelium and that the JE was maintained for at least 3 months after the eruption of the bioengineered tooth.

## Results

### The JE attached to the bioengineered tooth was derived from the odontogenic epithelium and was maintained for 3 months

**Figure 1:**
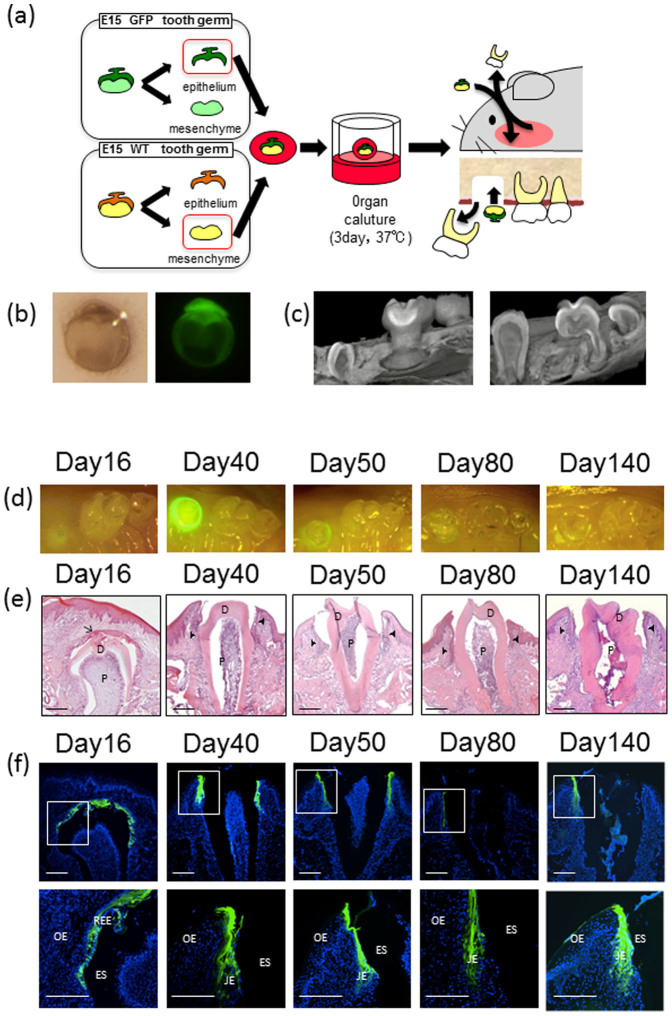
The JE attached to the bioengineered tooth was derived from the odontogenic epithelium. (a) Schematic representation of the generative technology of the bioengineered tooth germ. This schematic was originally drawn by one of the authors, Dr. Sara Yajima-Himuro. (b) Phase-contrast and GFP images of an organ-cultured bioengineered tooth germ on day 3. (c) Micro-CT images of the maxillary molar region immediately after eruption (30 days after transplantation) and full occlusion (50 days after transplantation). (d)–(f) Oral photographs, histological analysis, and fluorescence images of the bioengineered tooth during the eruption process, including before the eruption (16 days after transplantation), during the eruption (40 days after transplantation), after the full occlusion (50 days after transplantation), 1 month after the eruption (80 days after transplantation), and 3 months after the eruption (140 days after transplantation). (d) Oral photographs of a bioengineered tooth during the eruption process. Immediately after the dissection of the maxillae, occlusal views were imaged using a stereoscopic fluorescence microscope. (e) Histological analysis of a bioengineered tooth during the eruption process. The frozen sections were cut using a cryomicrotome (Microm) at a 6-μm thickness in the buccal-lingual direction. The sections were stained with hematoxylin and eosin (HE). D: dentine, P: pulp, arrow: reduced enamel epithelium, arrowhead: junctional epithelium (scale bar, 100 μm). (f) Fluorescence images of a bioengineered tooth during the eruption process. The lower row represents higher magnifications. Sixteen days after transplantation, GFP fluorescence was distributed only throughout the enamel epithelium of the bioengineered tooth. The primary JE showed green fluorescence 40 days after transplantation. After full occlusion (50 days after transplantation), the junctional epithelium showed GFP fluorescence that persisted until 140 days; however, the fluorescence intensity became weaker on the 140th day compared with that on the 80th day. Green: GFP, Blue: DAPI, REE: reduced enamel epithelium, JE: junctional epithelium, OE: oral epithelium, ES: enamel space (scale bar, 200 μm).

The reconstituted tooth germ, which consisted of green fluorescence protein (GFP)-transgenic mouse-derived epithelial cells and normal mouse-derived mesenchymal cells, was cultured three-dimensionally for 3 days, and the epithelial component exhibited green fluorescence (Fig. 1a,b). Subsequently, a single bioengineered molar tooth germ was transplanted into the bone hole formed by the extraction of the upper first molar region. The tooth germ structure was observed in the alveolar bone of the mice 16 days after transplantation, and the GFP fluorescence was distributed in the enamel epithelium of the bioengineered tooth but not in the odontoblast, dental pulp, or periodontal ligament, which is differentiated from the dental papilla (Fig. 1c–f). The cusp tip of the bioengineered tooth appeared in the oral cavity 30 days after transplantation. Forty days after transplantation, the reduced enamel epithelium fused with the oral epithelium that was partially attached to the enamel and initially formed the primary JE (Fig. 1d–f). The primary JE exhibited green fluorescence, which indicates that the primary JE was derived from the odontogenic epithelium (Fig. 1f). The reduced enamel epithelium gradually became shorter as the tooth erupted (Fig. 1d–f). Fifty days after transplantation, the bioengineered tooth finally reached the plane of occlusion, and the completely formed JE still exhibited green fluorescence at 140 days, although the intensity of fluorescence became weaker at 140 days than at 80 days (Fig. 1c–f). Therefore, the JE was maintained for at least 3 months without being replaced by the oral epithelium.

### The bioengineered tooth reproduced normal tooth development

**Figure 2:**
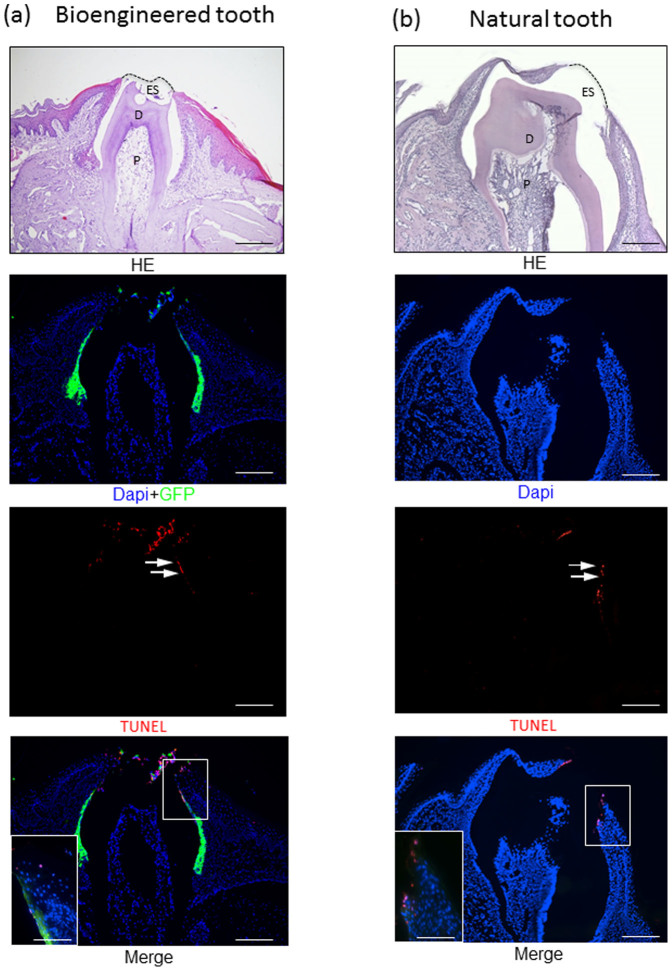
Apoptotic cells were detected at the top of the reduced enamel epithelium attached to the bioengineered tooth 30 days after transplantation using TUNEL assays. Apoptotic cell staining was conducted using the *In situ* Cell Death Detection Kit (Roche), according to the manufacturer's instructions, on the bioengineered tooth (a) and natural tooth (b) (arrow). Green: GFP, blue: DAPI, red: TUNEL, JE: junctional epithelium, OE: oral epithelium, ES: enamel space (scale bar, 100 μm).

**Figure 3:**
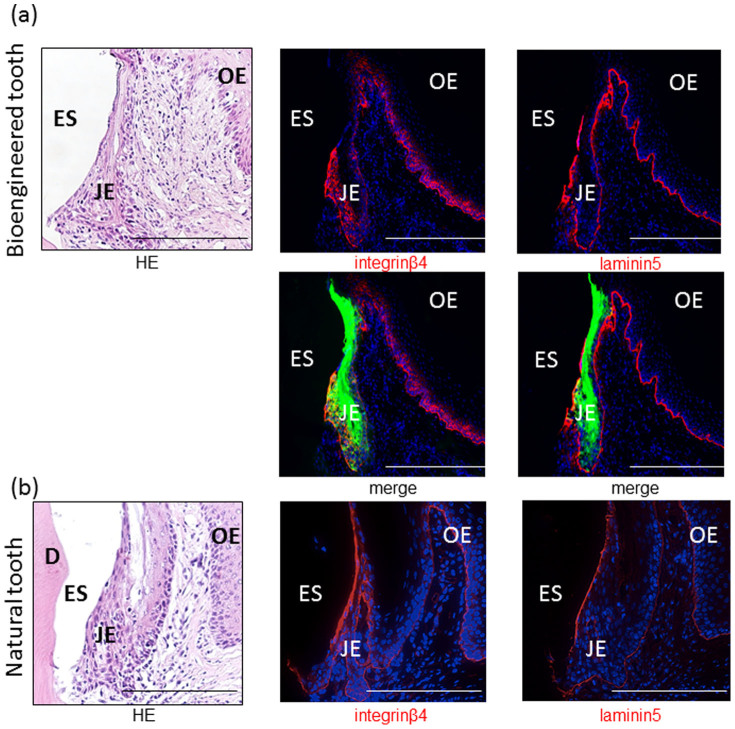
The bioengineered tooth-derived JE yielded integrin β4 and laminin 5 expressions similar to those of a normal tooth. The expression of integrin β4 and laminin 5 was detected in the bioengineered tooth 80 days after transplantation (a) and in the normal erupted tooth (b) by immunofluorescence analysis. Integrin β4 was found in the cytoplasm of three to four cell layers in the JE. Laminin 5 was found in the internal basal lamina of the junctional epithelium attached to the bioengineered tooth. The expression of integrin β4 and laminin 5 in the normal erupted tooth showed a similar distribution to that in the bioengineered tooth. Green: GFP, blue: DAPI, red: laminin 5 and integrin β4, JE: junctional epithelium, OE: oral epithelium, ES: enamel space (scale bar, 200 μm).

Next, to clarify whether the bioengineered tooth-derived JE reproduced the normal development of the JE, we assessed the presence of apoptotic cells in the reduced enamel epithelium of the bioengineered tooth using the TUNEL (terminal deoxynucleotidyl transferase dUTP nick-end labeling) assay because some of the reduced ameloblasts are removed by apoptosis, with the remainder constituting the JE in the normal mouse (Fig. 2b)^22^. A few TUNEL-positive apoptotic cells were detected in the reduced enamel epithelium of the bioengineered tooth (Fig. 2a). In addition, we detected the expression of laminin 5 and integrin β4, which are expressed in the normal JE^23^. Immunoreactivity to laminin 5 was found in the internal basal lamina of the sulcus epithelium attached to the bioengineered tooth and the basement membrane of the oral epithelium (Fig. 3a). This distribution is similar to that in a normal tooth (Fig. 3b). In addition, immunoreactivity to integrin β4 was observed in the cytoplasm of three to four layers of cells in the JE that were attached to the bioengineered tooth (Fig. 3a). This distribution was also similar to that in a normal tooth (Fig. 3b). Therefore, these results suggested that the bioengineered tooth reproduced normal tooth development.

### The bioengineered tooth-derived JE exhibited self-renewal potential

**Figure 4:**
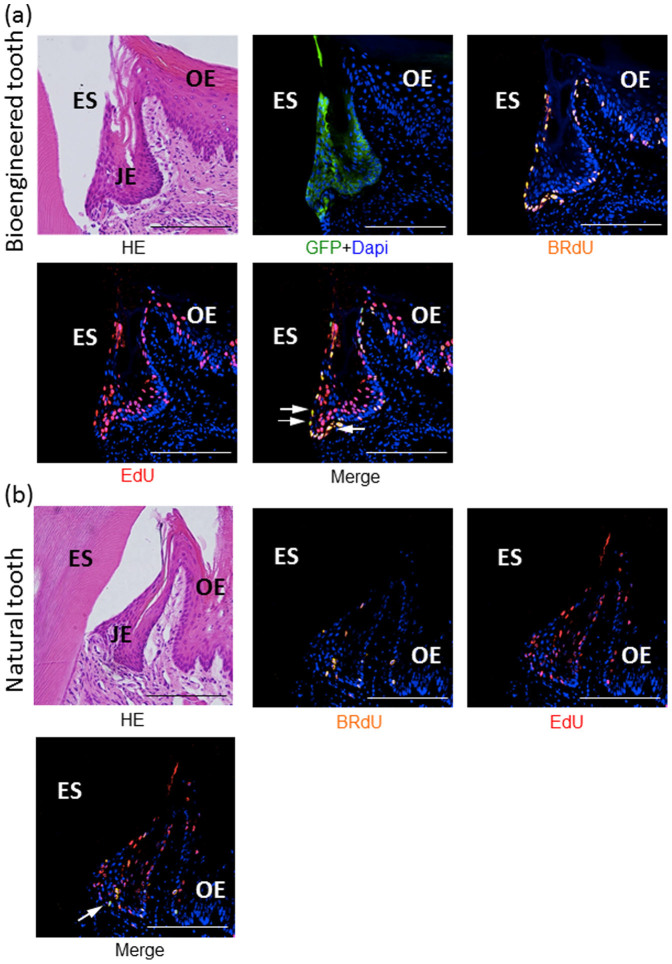
Double-labeling experiments using EdU and BrdU. Eighty days after transplantation, EdU was injected intraperitoneally into the mice, followed by the intraperitoneal administration of BrdU after 2 days. EdU-positive cells were found in the basal and supra-basal layers in the bioengineered tooth, whereas cells double-positive for EdU and BrdU were found in a few basal-layer cells (a). The distribution was similar between the bioengineered tooth and normal erupted tooth (b) (arrow). Green: GFP, blue: DAPI, red: EdU, orange: BrdU, JE: junctional epithelium, OE: oral epithelium, ES: enamel space (scale bar, 200 μm).

The JE has been reported to be maintained by a balance between epithelial cell proliferation and the exfoliation of cells through the gingival sulcus^24^. In addition, whether the JE is eventually replaced by the oral epithelium remains controversial^16^,^25^,^26^. Therefore, to examine whether the reduced enamel epithelium-derived JE of the bioengineered tooth had proliferative ability for a longer period, we performed double-labeling experiments using 5-ethynyl-2′-deoxyuridine (EdU) and 5-bromo-2′-deoxyuridine (BrdU). Consequently, there were a few BrdU-positive cells in the basal layer, and several EdU-positive cells were observed not only in the basal layer but also in the supra-basal layer (Fig. 4a,b). Interestingly, only a few cells in the basal layer showed positivity for both EdU and BrdU. Therefore, the bioengineered tooth-derived JE possessed self-renewal potential and may have contained epithelial stem-like cells.

## Discussion

The primary JE is believed to be formed by the fusion of the reduced enamel epithelium with the oral epithelium and gradually replaced by the oral epithelium^6^,^13^,^27^,^28^. Two proteins, odontogenic ameloblast-associated protein and amelotin, which have the potential to create the enamel, have been identified in the JE and observed during the formation and regeneration of the JE^17^,^18^,^19^. In addition, cytokeratin 19, which is a specific marker for the odontogenic epithelium, has also been identified in the human JE^29^,^30^,^31^. These findings suggest that the JE originates from the odontogenic epithelium. Interestingly, a mouse model expressing a truncated form of ameloblastin exhibited dental and JE defects because ameloblastin is expressed in ameloblasts, which are odontogenic epithelial cells^32^. Collectively, these findings indicate the possibility that the JE originates from the odontogenic epithelium of the erupted tooth, but more direct evidence is needed to confirm this possibility.

Bioengineered tooth methods have been reported to be successful, fully functioning tooth replacements in adult mice, achieved through the transplantation of the bioengineered tooth germ into the alveolar bone in the lost tooth region. The erupted and occluded bioengineered tooth displayed the correct tooth structure, hardness of mineralized tissues for mastication, and response to noxious stimulation, such as mechanical stress and pain, in cooperation with the other oral and maxillofacial tissues. Therefore, the bioengineered tooth model used in the present study is quite suitable for this purpose because it is possible to monitor only the odontogenic epithelium in the tooth germ using GFP in this model. This model has been established as a reproducible model for monitoring normal tooth eruption^33^.

Consistent with this model, our results demonstrated that the bioengineered tooth-derived reduced enamel epithelium fused with the oral epithelium similar to the process that occurs in the normal erupting tooth. Moreover, a few cells in the epithelium exhibited apoptosis. Thus, the reconstructed tooth reproduced normal tooth development. Furthermore, the bioengineered tooth-derived JE expression of laminin 5 and integrin β4, as well as their expression pattern, was similar to that in the normal JE^23^,^34^.

The turnover of the normal JE is much faster than that of the oral epithelium, in mice^23^,^35^. In the present study, BrdU was detected in the parabasal cells of the oral epithelium and the tips of the bioengineered tooth-derived JE, as well as in the normal JE. These findings are additional evidence that the turnover of the JE is much faster than that of the oral epithelium. The proliferation assay for the bioengineered tooth-derived JE and the normal JE revealed that there were a few cells that were double-positive for EdU and BrdU. Moreover, we demonstrated that the JE was derived from the odontogenic epithelium and maintained for at least 3 months. Therefore, we expected that the JE may have epithelial stem-like cells and self-renewal potential. Consequently, immunofluorescence staining of PCNA and p63, which have been demonstrated to be potential markers of oral keratinocyte stem cells, was performed. p63 staining was detected in the basal and superficial layer of the JE. In addition, PCNA staining was detected in the basal cells in the JE (Figure S1). These results are consistent with those of previous reports^24^,^36^. However, it is difficult to assess whether the JE possesses self-renewal potential and contains epithelial stem cells using only this experiment. Other experiments, such as lineage tracing method and such reconstruction assays, in which the fluorescent JE is surgically removed on one side of the toot, should be needed in futher studies^37^.

The JE, which is originally derived from the reduced enamel epithelium, has been proposed to potentially be replaced over time by JE that is formed by basal cells originating from the oral gingival epithelium^16^. The cells directly attached to the tooth are well known to have the potential to migrate toward the crown side and adhere. However, the source of these cells is unclear, and determining whether the JE is replaced by the oral epithelium is difficult^38^,^39^. To clarify whether the JE is maintained as the odontogenic epithelium or replaced by the oral epithelium, the re-formation of the JE following gingivectomy has been studied^25^,^40^,^41^,^42^,^43^. However, in the gingivectomy model, neglecting the existence of residual JE is difficult. Thus, the gingivectomy model has limitations in clarifying the replacement of the JE by the oral gingival epithelium.

In the present study, we demonstrated that the JE formed by the reconstructed tooth was maintained for at least 3 months and was not replaced by the oral gingival epithelium. We demonstrated that the JE initially originated from the odontogenic epithelium. However, these data cannot necessarily prove that the odontogenic epithelium-derived JE is maintained for a lifetime. For example, the time-dependent reduction of GFP in the bioengineered tooth-derived JE may indicate the partial replacement of the JE by the oral epithelium. Therefore, to clarify whether the odontogenic epithelium is maintained in the mature JE, further investigation is needed, such as the transplantation of a bioengineered tooth derived from a normal mouse into a GFP mouse. In addition, based on our present results, the JE in humans is expected to also be derived from the odontogenic epithelium. However, some structural differences exist in the JE between mice and humans. For example, there is no crevicular gingiva in the JE of humans, but a crevicular gingiva can be found in mice^44^. Therefore, more careful consideration is needed to assess JE development in humans.

## Methods

### Animals

C57BL/6 and C57Bl/6-Tg (CAG-EGFP) mice were purchased from CLEA Japan Inc. (Tokyo, Japan). The animal care and experimental procedures were approved by the International Animal Research Committee of Showa University in accordance with Japanese Government Law No. 105.

### Reconstitution of a bioengineered tooth germ

Molar tooth germs were dissected from the mandibles of ED14.5 C57BL/6 and C57Bl/6-Tg (CAG-EGFP) mice^20^,^33^ (Fig. 1a). The isolated tooth germs were incubated in 1.2 U/ml dispase (Roche) for 10 min at room temperature. The epithelium and mesenchymal tissues were separated using a fine needle. The mesenchymal tissues of the C57BL/6 mice were placed into a 30-ml gel drop of Cellmatrix type I-A (Nitta gelatin), and the epithelial tissues of the C57BL/6-Tg (CAG-EGFP) mice were then placed on the mesenchymal tissues. The bioengineered tooth germs were placed on a cell culture insert (0.4-mm-diameter pore, BD) and incubated at 37°C in a humidified atmosphere at 5% CO_2_. The reconstituted explants were cultured for 3 days on cell culture inserts in 6-well culture plates (BD) in Dulbecco's modified Eagle's medium (Sigma) supplemented with 10% fetal bovine serum (FBS).

### Transplantation

The upper first molars of 3-week-old C57BL/6 mice were extracted under deep anesthesia. The tooth extraction sites were allowed to be repaired in the mice for 3 weeks (Fig. 1a). Subsequently, an incision of approximately 1.5 mm in length was made through the oral mucosa at the extraction site. A fine pin vise (Tamiya) was used to create a bony hole of approximately 0.5–1.0 mm in diameter in the exposed alveolar bone surface. Immediately before transplantation, we removed the collagen gel from the bioengineered tooth germ, and the explants were then transplanted into the bony hole in the right direction. The incised oral mucosa was then sutured with 8-0 nylon (Bear Medoc Corp.). The mice containing the transplants were fed a powdered diet until the regenerated tooth erupted.

### Micro-CT

An X-ray analysis was performed on the upper jaws of the mice that received a transplanted bioengineered tooth using a micro-CT device (inspeXio SMX-90CT, Shimadzu, Kyoto, Japan) with exposures at 90 kV and 110 mA.

### Histological and immunofluorescence analysis

The maxillae were dissected and fixed with 4% paraformaldehyde for 6 h at 4°C. After decalcification with 10% ethylenediaminetetraacetic acid (EDTA) for 2 weeks at 4°C, the specimens were embedded in optimal cutting temperature compound (Sakura) and then immediately snap-frozen in liquid nitrogen-cooled isopentane. The frozen sections were cut using a cryomicrotome (Microm) at 6-μm thickness in the buccal-lingual direction. The sections were stained with hematoxylin and eosin (HE) or were used for immunofluorescence staining. For immunofluorescence staining, the frozen sections were air-dried for 10 min, washed with Tris-buffered saline (TBS), and pre-incubated with blocking solution (Dako) for 10 min. The sections were incubated with an anti-integrin β4 goat polyclonal antibody (Cat. No. AF3059; 1:100 dilution; R&D Systems) and an anti-laminin 5 rat monoclonal antibody (Cat. No. ab105472; 1:100 dilution; Abcam) for 2 h at room temperature. After washing in TBS, the sections were incubated for 1 h at room temperature with an anti-rabbit IgG antibody conjugated with Alexa 594 or an anti-rat IgG Alexa 594 of donkey origin (1:200 dilution; Molecular Probes). After counterstaining with 4′, 6-diamidino-2-phenylindole dihydrochloride (DAPI; 1:5000 dilution; Dojindo), all specimens were examined and photographed (Nikon A1 Confocal Microscope System).

### TUNEL staining

Apoptotic cell staining was conducted using the *In situ* Cell Death Detection Kit (Roche) according to the manufacturer's instructions.

### Proliferation assay

To measure the kinetics of the bioengineered tooth-derived JE proliferation, EdU was injected intraperitoneally into the mice at 10 μg/g, followed by intraperitoneal administration of BrdU at 30 μg/g after 3 days. EdU staining was performed using the Click-iT™ EdU imaging kit (Invitrogen) according to the manufacturer's instructions. BrdU was detected using a FITC-conjugated mouse monoclonal antibody directed against BrdU (Roche) according to the manufacturer's instructions.

## Author Contributions

S.Y. designed the experiments, performed most of the experimental work, and co-wrote the manuscript. The bioengineered tooth germ was reconstructed by Miho O. Masamitsu O. performed the transplantation of the bioengineered tooth. G.Y. aided in the design of some experiments. K.N., J.T. and M.F. analyzed the data. K.M., Tetsuhiko T. and Takashi T. supervised the project. M.Y. designed the experiments, supervised the project, and wrote the manuscript.

## Supplementary Material

Supplementary Information Supplementary Information
