# Supplementary material for: The junctional epithelium originates from the odontogenic epithelium of an erupted tooth
Source: Sci Rep. 2014 May 2;4:4867. doi: 10.1038/srep04867 (PMC4007090; doi:10.1038/srep04867)
Supplement: Supplementary Information [file srep04867-s1.pdf]

Supplementary Information

The junctional epithelium originates from the odontogenic epithelium of an erupted tooth

Sara Yajima-Himuro[1], Masamitsu Oshima[2], Gou Yamamoto[4], Miho Ogawa[3], Madoka Furuya[1], Junichi Tanaka[4], Kousuke Nishii[1], Kenji Mishima[4], Tetsuhiko Tachikawa[4], Takashi Tsuji[2,3], Matsuo Yamamoto[1]

[1]Department of Periodontology, Showa University School of Dentistry, 2-1-1 Kitasenzoku, Ohta-ku, Tokyo 145-0062, Japan

[2]Research Institute for Science and Technology, Tokyo University of Science, Chiba 278-8510, Japan

[3]Organ Technologies Inc., Tokyo 101-0048, Japan

[4]Division of Pathology Department of Oral Diagnostic Sciences, School of Dentistry, Showa University School of Dentistry, 1-5-8, Hatanodai, Shinagawa-ku, Tokyo, 145-8515, Japan

Correspondence and requests for materials should be addressed to M.Y. (yamamoto-m@dent.showa-u.ac.jp).

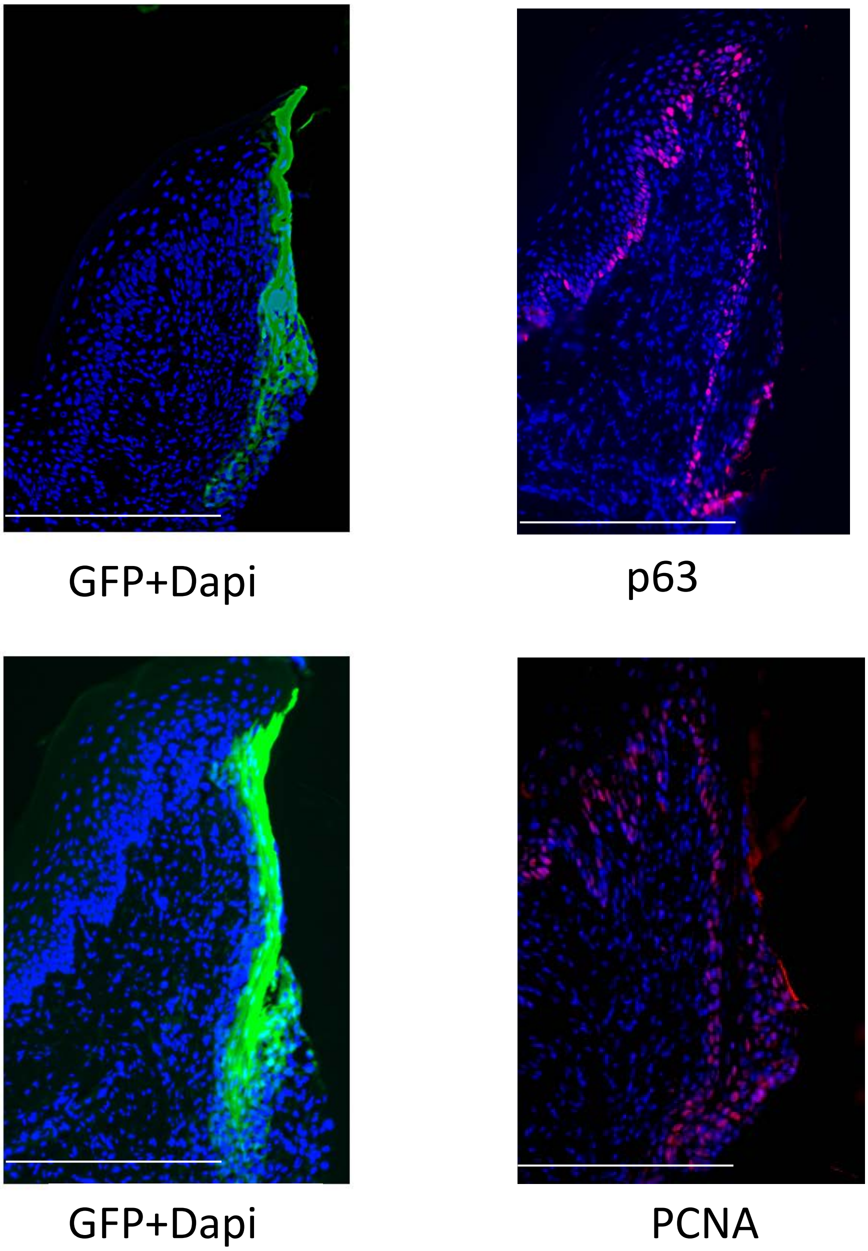

FIGURE S1: p63 staining was detected in the basal and superficial layer of the JE. In addition, PCNA staining was detected in the basal cells in the JE. (scale bar, 200 μm)

ADDITIONAL MATERIALS AND METHODS

The maxillae were dissected and fixed with 4% paraformaldehyde for 6 h at 4°C. After decalcification with 10% ethylenediaminetetraacetic acid (EDTA) for 2 weeks at 4°C, the specimens were embedded in optimal cutting temperature compound (Sakura) and then immediately snap-frozen in liquid nitrogen-cooled isopentane. The frozen sections were cut using a cryomicrotome (Microm) at 6-μm thickness in the buccal-lingual direction. The sections were used for immunofluorescence staining. For immunofluorescence staining, the frozen sections were air-dried for 10 min, washed with Tris-buffered saline (TBS), pretreated using a citrate buffer, PH 6.0, and pre-incubated with blocking solution (Dako) for 10 min. The sections were incubated with an anti- p63 rabbit polyclonal antibody (Cat. No. ab53039; 1:200 dilution; Abcam) and an anti-PCNA mouse monoclonal antibody (Cat. No. 610664; 1:100 dilution; BD Transduction Laboratories) for 1 h at room temperature. After washing in TBS, the sections were incubated for 1 h at room temperature with an anti-rabbit IgG antibody conjugated with Alexa 594 or an anti-mouse IgG Alexa 594 of donkey origin (1:200 dilution; Molecular Probes). After counterstaining with 4’, 6-diamidino-2-phenylindole dihydrochloride (DAPI; 1:5000 dilution; Dojindo), all specimens were examined and photographed (Nikon A1 Confocal Microscope System).
